# Simultaneous Extraction of Oil and Protein from Silkworm (*Bombyx mori* L.) Pupae (Lueng Parroj var.) and Their In Vitro Skin Moisturization

**DOI:** 10.3390/molecules28207032

**Published:** 2023-10-11

**Authors:** Pannarasi Susirirut, Natthawut Thitipramote, Phanuphong Chaiwut

**Affiliations:** 1 School of Cosmetic Science, Mae Fah Luang University, Muang, Chiang Rai 57100, Thailand; taooan@gmail.com (P.S.); natthawut.thi@mfu.ac.th (N.T.); 2 Green Cosmetic Technology Research Group, School of Cosmetic Science, Mae Fah Luang University, Muang, Chiang Rai 57100, Thailand

**Keywords:** simultaneous extraction, silkworm pupae, oil, protein, moisturization

## Abstract

Oil and protein from silkworm (*Bombyx mori* var. Leung Pairoj) pupae, by-product from sericulture, were extracted and evaluated for their potential uses as skin biomoisturizer. The silkworm pupae (SWP) oil and protein were simultaneously extracted by using three-phase partitioning (TPP) method and determined for their physicochemical properties including fatty acid and amino acid content, respectively. The highest yields of oil and protein at 8.24 ± 0.21% and 8.41 ± 0.26% *w*/*w*, respectively were obtained from 18 h extraction. Fatty acid analysis of SWP oil was rich in linolenic acid (37.81 ± 0.34%), oleic acid (28.97 ± 0.13%), palmitic acid (21.27 ± 0.05%), stearic acid (6.60 ± 0.09%) and linoleic acid (4.73 ± 0.21%). The clear yellow SWP oil possessed saponification value of 191.51 mg/g, iodine value of 119.37 g I_2_/g and peroxide value of 2.00 mg equivalent O_2_/kg. The SWP protein composed of 17 amino acids which aspartic acid, glutamic acid, glycine and serine were the major residues. SDS-PAGE analysis revealed that the SWP protein consisted of distinct protein at around 51, 70, 175 and over 175 kDa. Cytotoxicity of the SWP oil and protein was evaluated by using MTT assay and they showed low cytotoxicity toward keratinocyte cell (HaCat cell line). The SWP oil provided moisturizing effect on pig skin comparable to olive oil, while 1% and 2% of SWP protein showed higher moisturizing efficacy than 3% hydrolyzed collagen. The study indicated that the SWP oil and protein could be potential biomoisturizers for cosmetic products.

## 1. Introduction

In sericulture or silk production, silk fiber is harvested by placing the cocoons in boiling water to unwound the silk fiber or fibroin from the cocoons. The silkworm pupae (SWP) are also produced in this reeling, and it is generally considered as waste to be removed [1]. The SWP contains 47.87–60.70% protein and 23.50–30.85% of fat [2,3]. Because of their large quantities of proteins, fats, carbohydrates and vitamins content, the SWP are currently utilized by human consumption [2,4], poultry feed [5] and aquafeed [6]. There have been also reported that the SWP proteins possessed a potential therapeutic target for human breast cancer [7], immunomodulating [8], antioxidant capacity [9] and Parkinson’s disease resistance [10]. Various studies have been revealed that the edible insect proteins provide emulsion capacity, water/oil absorption ability and gel-forming ability [11] indicating that the pupae protein is good functionality in cosmetic products. However, there a few studies reporting cosmetic application of SWP.

The proteins and lipids are considered as skin moisturizers. The proteins use hygroscopic property to absorb moisture from environment to keep skin more hydrated, while the lipids provide emollient property that they are absorbed into corneocyte intercellular to provide skin barrier function and prevent trans epidermal water loss. Both proteins and lipids improve skin softness, smoothness, flexibility and reduce wrinkle and melanin production [12]. One aspect of healthy skin is the preservation of the skin barrier, consisting of protein-rich corneocytes surrounded by organized intercellular lipids [13]. In cosmetic industry, the proteins and oils are obtained from plant and animal. Soy and wheat proteins are good examples of plant proteins, while jojoba oil and olive oil have been extensively used in cosmetic industry. Animal proteins e.g., hydrolyzed collagen and hydrolyzed keratin and animal fate like tallow are also widely utilized as natural moisturizing agents. There is limited information of the use of protein and oil from insect, especially from SWP, in cosmetic application. Therefore, it is worth to investigate extraction and utilization the oil and protein from the SWP as alternative skin moisturizing agent in cosmetic application.

Traditionally, proteins and oils from one source of plant or animal are extracted separately step by step. Oil and lipids can be extracted by using mechanical methods such as maceration or cold-press extraction as well as chemical extraction including solvent extraction with reflux or CO2 extraction etc. [14]. Even the solvent extraction is widely accepted for oil extraction [15], modern extraction techniques for example microwave-assisted extraction or sonicated-assisted method have become more interest. Thereafter, the protein extraction could be carried out by using various downstream processing such as precipitation by salts or solvents, filtration or centrifugation, chromatographic separation and lyophilization. Alternative methods like aqueous two phase system and three phase partitioning (TPP) have been introduced due to their simplicity and scalability. Each method has its unique benefits and disadvantages based on their efficiency and economy. Various extraction methods are successfully performed in laboratory scale but have limitation on industrial scale.

The TPP is an interesting method for biomolecules separation. This method is an economic protocol which it can use crude suspension directly, simple procedure with short processing time [16]. It also does not denature the protein because the process is done at the ambient condition. The TPP has been widely applied in the recovery of active compounds from plant sources and algae such as proteins and enzymes [15,17], lipid [18,19], polysaccharide [20] and other bioactive compounds e.g., andrographolide, curcuminoids, mangiferin and oleoresin. The TPP mainly separates oil in upper phase, proteins in the middle precipitate phase and some hydrophilic compounds like polysaccharides in the bottom aqueous simultaneously [21].

In the light of this, it is worthwhile to use it as a new economical technique for simultaneous separation of proteins and oils from the SWP which is mass by-product from sericulture. In this study, the TPP was used for the separation of oil and proteins from SWP via a single-step extraction. The extracted oil and protein were investigated for their properties and moisturization efficacy on pig skin.

## 2. Results

### 2.1. Simultaneous Extraction of Oil and Protein from SWP

The SWP used in this study was Lueng Pairoj variety which its color before drying was golden yellow and changed to yellowish-brown after drying possessing 20.18 ± 3.15% (*w*/*w*) dried weigh. The TPP method was used in the single-step extraction to concurrently obtain the lipid at the top phase and the protein at the interphase. As shown in Table 1, the highest extractive yields of oil and protein of 8.24 ± 0.21% *w*/*w* and 8.41 ± 0.26% *w*/*w* were obtained from 18 h of extraction. The result showed that increase in extraction duration resulted as the oil and protein yield increase. The yield of protein yield obtained from the TPP in this study was considerable. Protein content in the protein interphase was also determined, and it was observed that the protein contents in the range of 267.36 ± 1.25–268.34 ± 1.71 mg/g extract were not significantly different among all extraction times as shown in Table 1.

In this study, it is noticed that the silkworm pupae powder sample was directly used in the TPP process, and the oil and protein could be simultaneously extracted. This was a potential technique for saving electric energy and time of sample preparation.

### 2.2. Fatty Acid Composition and SWP Oil Properties

Unsaturated fatty acids with 18 carbon alkyl chains were found to be major components in the SWP oil accounted for 71.51% *w*/*w*. The highest concentration of 37.81 ± 0.34% *w*/*w* was that of alpha-linolenic acid, followed by oleic acid with a concentration of 28.97 ± 0.13% *w*/*w.* Palmitic acid was the highest content among saturated fatty acids accounted for 21.27 ± 0.05% *w*/*w*, whereas myristic acid and lauric acid were revealed as minor compounds (Table 2).

Physical and chemical properties of silkworm pupae oil were presented in Table 3. The oil appearance was clear viscous yellow with a unique smell of insect oil. Saponification value of 191.51 ± 0.22 mg KOH/g implied the oil containing high amount of unsaturated fatty acids. The iodine value of silkworm pupae oil was 119.37 ± 0.67 g I_2_/100 g which was quite relatively high values related to the high unsaturated fatty acid components. Low number of acid value in silkworm pupae oil implied high quality of oil. Peroxide value shows the initial evidence of rancidity in unsaturated fats and oils. The silkworm pupae oil exhibited 2.66 mEq O_2_/kg oil.

### 2.3. SDS-PAGE of SWP Protein

**Figure 1:**
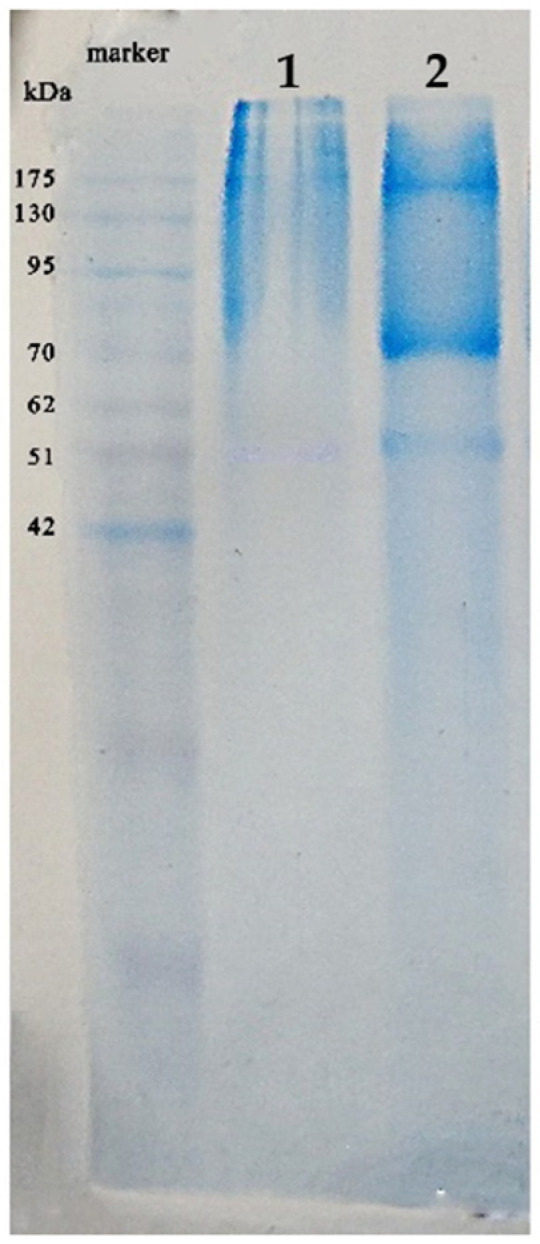
SDS-PAGE profile of silkworm pupae protein (15 µg loaded), lane 1; water extract of silkworm pupae and lane 2; TPP silkworm pupae protein.

Protein pattern and molecular weight distribution of the SWP protein were analyzed by using SDS-PAGE consisting of 10% separating gel and the result is shown in Figure 1. After TPP extraction, distinct protein bands around 51, 70, 175 kDa as well as high intensity band over 175 kDa were observed. There was also presence low intensity band of protein at lower 42 kDa region. The water extract of silkworm pupae (lane 1) might contain various kinds of protein distribution which exhibited low intensity. The TPP was apparently exhibited its high potential to extract and concentrate protein from the crude silkworm pupae extract. Differences in molecular size of protein extracted from silkworm pupae was probably from variety differences and the method of extraction.

### 2.4. Amino Acid Composition of SWP Protein

The SWP protein contained both essential and non-essential amino acids, as shown in Table 4. The amino acid composition of the SWP protein extracted by the TPP method was 25.12 g/100 g. Major amino acid components were aspartic acid, glycine, glutamic acid and serine, respectively. Amino acids content in the skin namely serine, glycine, alanine and threonine are one of natural moisturizing factors (NMFs).

### 2.5. Cell Cytotoxicity of SWP Oil and Protein

**Figure 2:**
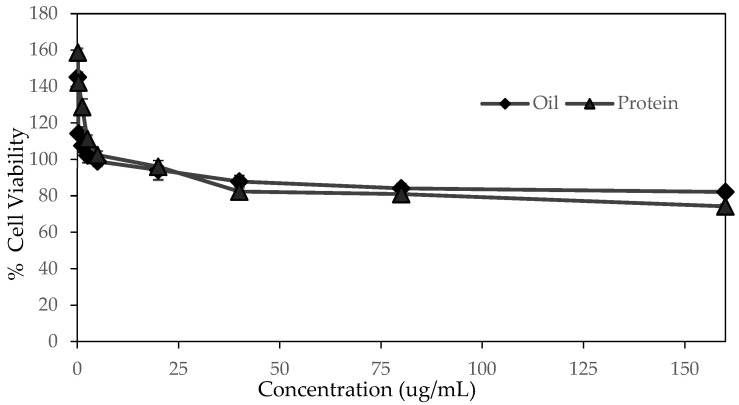
Cytotoxicity evaluation of silkworm pupae oil and protein.

The MTT test for cell line cytotoxicity of silkworm pupae oil and protein are shown in Figure 2. The silkworm pupae oil showed no cytotoxicity on HaCAT cell at doses tested ranging from 0.156–160 µg/mL. The silkworm pupae protein was proven to be safe at concentration ranging 0.156–80 µg/mL. These results suggested that the protein and oil from SWP could be considered safe for application as cosmetic ingredients. Safety assessment in cell culture models is important for topical products.

### 2.6. In Vitro Moisturizing Effect of SWP Oil and Protein

**Figure 3:**
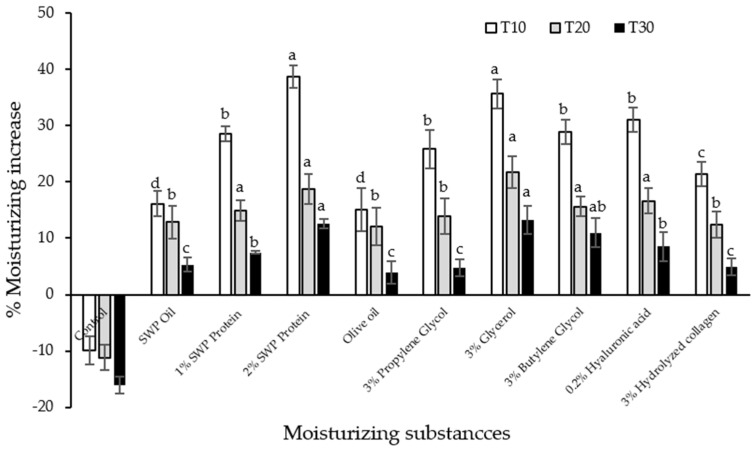
Moisturization efficacy of silkworm pupae oil and protein compared to commercial cosmetic moisturizers. Control was skin without application of moisturizing substances. Differencet letters indicate significant difference (*p* ≤ 0.05) at the same time measurement.

A study was designed to test the efficacy of pupae oil and protein compared to commercial moisturizers, including olive oil, glycerol, propylene glycol, butylene glycol, hyaluronic acid and collagen. Concentration of each substance was used according to their effectiveness from previous reports. Figure 3 shows skin moisturizing effects of the pupae oil and protein compared to commercial cosmetic moisturizers. At 10 min after application (T10), 2% SWP protein and 3% glycerol were found to be the highest skin moisturizing effect at 38.75% and 35.68%, respectively. However, these moisturizing effects was not significant difference between them. Moisturizing efficacy of all substances tested decreased by time. At T30, the efficacy of 2% SWP protein declined to 12.60% which were comparable to 3% glycerol and 3% butylene glycol and higher than 0.2% hyaluronic acid, 3% hydrolyzed collagen, 3% propylene glycol and olive oil, respectively. The SWP oil possesses 16.12% moisturizing increase at T10 and it was not significantly different to olive oil. The results indicated that SWP oil and protein could be effective moisturizers for cosmetic products.

## 3. Discussion

### 3.1. Simultaneous Extraction of Oil and Protein from SWP

The TPP is an economic method because it is a simple procedure with short processing time and use inexpensive chemicals with mild condition and do not denature proteins [22]. The extracted protein is concentrated and can apply for mass production. It can extract oil and proteins at the same time and does not denature the proteins, due to the process is performed at the ambient condition and provide high protein extract [15]. The TPP has been previously used to extract aloe polysaccharide and protein simultaneously making it be single-step extraction [20].

The yield of protein in this study increased from 2.56 ± 0.36% to 8.41 ± 0.26% when the extraction time raised from 3 to 18 h. There was reported that blending frozen silkworm pupae (WSPB variety) with cold water at 4 °C provided the lower protein yield of 3.96% *w*/*w* [23]. Even the protein yields were increased upon the extraction duration, the protein contents were quite constant. These might be explained by other chemical such as chitin was more extracted when extraction time rise. There was reported that soxhlet and maceration extractions exhibited the SWP oil yields of 24.00–28.98% and 3.81–7.00%, respectively [24].

### 3.2. Fatty Acid Composition and SWP Oil Properties

Palmitic acid was the highest content among saturated fatty acids accounted for 21.27 ± 0.05% *w*/*w*, whereas myristic acid and lauric acid were revealed as minor compounds (Table 2). These results were consistent with previous report that the silkworm (None-Ruesee variety) pupae oil obtained from maceration extraction provided alpha-linolenic acid as the major fatty acid of 42.31% followed by oleic acid as 30.17% [24]. However, the oil obtained from silkworm (Khewsakon varieties) pupae showed oleic acid as the highest component followed by alpha-linolenic acid [24]. The SWP oil usually composed mainly 70% of 4 kinds unsaturated fatty acids (linoleic, alpha-linolenic, oleic acids and less than 1% palmitoleic acids) and mainly 2 kinds of saturated fatty acids (palmitic and stearic acids) [25].

Lipids and oils function to hydrate and improve skin softness, flexibility, smoothness, and lubricity. Among the various kinds of fatty acid, linoletic, strearic and palmitic acid are most common used in moisturizer [26]. Long-chain fatty acids, for example, stearic, oleic and linoleic acids are emollient influencing in skin physiology and pathology via their effects on skin barrier functions, eicosanoid production, membrane fluidity, and cell signaling [27]. Supae oil provided various kinds of long-chain fatty acids especially the C18 length. Thus, the moisturizing property of silkworm pupae oil can be well utilized in a moisturizing product.

The oil appearance was clear viscous yellow with a unique smell of insect oil. Saponification value of 191.51 ± 0.22 mg KOH/g implied the oil containing high amount of unsaturated fatty acids. This was similar to safflower seed oil possessing 190.23 mg KOH/g respectively [28].

The iodine value of silkworm pupae oil was 119.37 ± 0.67 g I_2_/100 g which was a relatively high value, attributed to its high unsaturated fatty acid components. The result was comparable to the iodine values of previous report that the five varieties of silkworm pupae oils extracted by using soxhlet method appeared to be between 100.20–128.01 g I_2_/100 g [24,29] which comparable to that of canola oil of 110–120. Low number of acid value in silkworm pupae oil implied high quality of oil. Acid value is defined as the weight of KOH in mg needed to neutralize the organic acids present in 1 g of fat [28,29]. Peroxide value shows the initial evidence of rancidity in unsaturated fats and oils. The silkworm pupae oil exhibited 2.66 mEq O_2_/kg oil which was comparable to 2.20 mEq/kg of palm oil [29]. The silkworm pupae oil exhibited 2.66 mEq O_2_/kg oil which was comparable to 2.20 mEq/kg of palm oil [29].

### 3.3. SDS-PAGE of SWP Protein

The TPP was apparently exhibited its high potential to extract and concentrate protein from the crude silkworm pupae extract. Protein profile of the SWP has previously been reported revealing protein bands ranging from lower 20 kDa to 250 kDa [22]. Supernatant of silkworm pupae extract treated with acidic buffer exhibited major protein bands around 14, 24, 30, 36 and 45 kDa [9]. Wang et al. [30] reported four major protein components in silkworm pupae, including albumin (97.4 kDa, 61.4 kDa, 44.4 kDa and 26.7 kDa), glutelin (200 kDa and 15 to 60 kDa), globulin (130.0 kDa and 26.8 kDa) and prolamin (15.3 to 46 kDa). Differences in molecular size of protein extracted from silkworm pupae was probably from variety differences and the method of extraction.

### 3.4. Amino Acid Composition of SWP Protein

Amino acid residues presented in the study were consistent with previous report that revealed some amino acids such as glutamic acid, aspartic acid, valine, isoleucine, methionine, phenylalanine and proline containing in silkworm pupae protein [31]. Result from this study showed aspartic acid was the highest component followed by glutamic acid. However, there were reports that the *B. mori* pupae contained higher glutamic acid than aspartic acid [32,33]. Amino acids content in the skin namely serine, glycine, alanine and threonine are one of natural moisturizing factors (NMFs) [34]. The skin NMFs made of a mixture of low molecular weight soluble hygroscopic substances such as lactic acid, pyrrolidone carboxylic acid, and amino acids which are the main functions for hydration of the stratum corneum [27]. Free amino acids make up to 40% of the NMFs. Glycine, proline and alanine are also major components of collagen. Moreover, various amino acids have been reported to possess skin and collagen regeneration promotion activity such as arginine, glutamine, and glycine. Cysteine, methionine, histidine and arginine have also been described as antioxidant and UV damage protection [34].

### 3.5. Cell Cytotoxicity of SWP Oil and Protein

The protein and oil from SWP could be considered safe for application as cosmetic ingredients. Safety assessment in cell culture models is important for topical products. Mixture of component present extracts can produce local reactions when applied to the skin, and this can be a crucial factor for use as cosmetic ingredients [35]. It was also found that maximum concentration of ethanolic extract of from silkworm pupae varieties Nangnoi Srisaket-1 and LuangSurin providing 90% survived cells were at 60 and 410 µg/mL, respectively [36]. Safety of silkworm oil and protein is also supported by its routine consumption in China, Japan, Thailand, Korea and Vietnam and the FAO reporting that the silkworm is one of the most promising species for industrial feed production [1].

### 3.6. In Vitro Moisturizing Effect of SWP Oil and Protein

Moisturizers are intrinsic to the function and beauty of the skin. They are used to improve the skin elasticity and aging which is determined by hydration level [37]. Skin moisturization is commonly measured using methods based on the electrical properties of the skin [38]. The moisturizing efficacy of the tested SWP oil and protein was evaluated on pig skin model. The selected pig skin model relies on several factors, including cost, availability, ease of handling, functional and structural similarity to humans [39]. A study was designed to test the efficacy of pupae oil and protein compared to commercial moisturizers, including olive oil, glycerol, propylene glycol, butylene glycol, hyaluronic acid and collagen. Concentration of each substance was used according to their effectiveness from previous reports.

It was observed that the moisturizing effect of SWP protein was significantly higher than that oil that may be related to the amino acid contents such as tryptophan, tyrosine and phenylalanine that highly attract water molecule for skin hydration [32,40] and might be from the capacitance principle of equipment measurement. Leelapornpisid et al. [40] reported the moisturizing effect on pig skin of aqueous extract from freshwater macroalga *Rhizoclonium hieroglyphicum* compared with glycerol, propylene glycol and hyaluronic acid. The pig skin was also used to investigate the moisturizing effect of a tea (*Camellia assamica*) seed oil, which consisted of oleic acid, linoleic acid and palmitic acid. It was found that the tea seed oil showed similar results to olive oil [41]. Mucilage from *Hibiscus mutabilis* Linn was also reported to exhibit moisturizing efficacy on pig skin similar to glycerol within 30 min testing [42].

Oils, lipid and large-molecular weight substances provide film on the skin surface to retard evaporation of water from inside. Lipids are also required for effective process of dead skin cell shedding to the environment in healthy skin [43]. Oleic acid function as lipid lamellar sheet stabilizer which can reduce the loss of water from the skin, especially in elderly people [43]. Hydrophillic substances including humectants can increase skin hydration by attracting water to the skin surface [44]. High-molecular weight hydrophiles like hyaluronic acids and protein including collagen can function in similar manner to create a blanket against water evaporation from skin and create an environment for skin hydration [44]. In healthy skin, good skin barrier is preserved by protein-rich corneocytes surrounded by organized intercellular lipids. Moisturizers function to reduce transepidermal water loss and creating an environment optimum for skin barrier function and maintenance of skin health [13]. Therefore, the SWP oil and protein provided moisturizing effects by creating a thin film over skin to prevent water loss from inside. The SWP protein also attracted water from environment to generate skin hydration. These properties were promise that the SWP oil and protein being a potential biomoisturizers for cosmetic application.

## 4. Materials and Methods

### 4.1. Chemicals

Ammonium sulfate ((NH_4_)_2_SO_4_) and tert-butanol were obtained from Merck (Darmstadt, Germany). Sodium dodecyl sulfate (SDS), bovine serum albumin (BSA), casein *L*-cysteine, beta-mercaptoethanol (*β*-ME) and Coomassie Brilliant Blue (CBB) G-250 and R-250 were acquired from Sigma-Aldrich (St. Louis, MO, USA). All chemicals and solvents used were analytical grade. Silkworm pupae (*Bombyx mori* L.) var. Lueng Pairoj (J108bivotinexNanglai: multivoltine), by-product from sericulture, were collected from The Queen Sirikit Department of Sericulture in Hua-Hin, Prachuapkhirikhan Province, Thailand. Glycerol, propylene glycol, butylene glycol, hydrolyzed collagen, hyaluronic acid and olive oil were cosmetic grade.

### 4.2. Preparation of SWP Samples

Thai variety silkworm pupae were washed with distilled water, air-dried, and dried in a tray dyer at 55 °C until their weight were constant which their humidity less than 10%. The dried pupae were ground and sieved through sieving material 60 mesh to obtain sample powder and stored at 4 °C for further testing.

### 4.3. Simultaneous Extraction of SWP Oil and Protein Using TPP

The extraction of oil and protein in this study using only single-step of the TPP which was modified from previous report of Pintathong et al. [45]. The extraction was carried out in 250 mL glass reactor provided with mechanical stirrer. The pupae powder was directly used without preliminary crude extract preparation. The system consisted of silkworm pupae powder, ammonium sulfate, *t*-butanol and water. The silkworm pupae powder was enveloped with a sheet cloth and then soaked in distilled water at the ratio 1:3 *w*/*v* for five min. Then ammonium sulfate to the final concentration of 30% *w*/*v* was added to the mixture. After the salt was dissolved, *t*-butanol was added to the vessel. The sample mixture was stirred with a magnetic stirrer for 3, 6, 12 and 18 h. The pupae residue in a sheet cloth envelop was removed and the mixture was transferred to a separating funnel to stand at ambient condition for 10 min. Three distinct phases were simultaneously occurred. The top layer of *t*-butanol containing lipids was subjected to evaporate the solvent with a rotary evaporator. After rotary evaporation, yield, fatty acid composition and oil properties were investigated. The middle phase of protein precipitate was dissolved in minimum amount of DI water and dialyzed for 12 h against DI water. After lyophilization, yield, protein content and amino acid composition of the lyophilized protein were determined.

### 4.4. Analysis of SWP Oil

#### 4.4.1. Determination of Fatty Acid Composition

Fatty acid composition of silkworm pupae oil was analyzed by using GCMS. The fatty acid methyl esters (FAMEs) were prepared by mixing of 3 g oil with 3.00 mL of 0.9 M H_2_SO_4_ in methanol and 1.00 mL of toluene [45]. The mixture was refluxed for 2 h at 75–80 °C and the solvent was removed by rotary evaporator at 40 °C. Recovered FAMEs sample was adjusted to pH 6. The GC-MS analysis was carried out with a system composed of 0.25 mm × 30 mm × 0.25 μm capillary column (Agilent 190915-43 HP-5MS) at 250 °C using helium as a carrier gas with 1.0 mL/min flow rate. The injector and detector temperature were set at 220 °C. The FAMEs was detected at 20–250 Da and identified by NIST08 database.

#### 4.4.2. Determination of Oil Properties

Color measurement of the silkworm pupae oil was performed by using Colorimeter (Hunter Lab, Miniscan XE plus, Hunter Associates Laboratory, Inc., Reston, VA, USA) and the CIE L*, a*, b* color scale was reported. The viscosity was measured by using a viscometer (Bookfield/RVD-II+P), 20 rpm and spindle No.2 at 30 °C. Peroxide value, acid value, saponification value and iodine value were determined according to the American Oil Chemists’ Society [46].

### 4.5. Analysis of SWP Protein

#### 4.5.1. Determination of Protein Content

Protein content of the TPP protein precipitate was measured by Bradford method [47] using bovine serum albumin (BSA) as protein standard.

#### 4.5.2. Determination of Amino Acid Composition

Determination of amino acids composition in silkworm pupae protein was performed according to AccQ Tag method using HPLC (Waters, 2690, Waters Corp., Milford, MA, USA) with a Waters AccQ Tag C18 column (3.9 mm × 150 mm) at the temperature of 37 °C. The samples of 10 µL were injected and their peaks were compared to standard amino acids.

#### 4.5.3. SDS-PAGE Analysis

SDS-PAGE of the samples was performed according to the method of Laemmli [48] with slight modification. Protein samples (5 and 15 μg) were loaded onto the electrophoresis gel (20 mA/gel) made of 4% stacking and 15% separating gels. Tris-HCl (0.5 M), pH 6.8 and Tris-HCl (1.5 M), pH 8.8 were used as stacking gel buffer and resolving gel buffer, respectively. Sample buffer consist of 0.025 M Tris, 0.192 M Glycine, 0.1% *w*/*v* SDS, 10% glycerol and 5% βME, pH 8.3. After electrophoresis, the gel was then stained overnight with staining solutions (0.02% (*w*/*v*) CBB R-250 in 50% (*v*/*v*) methanol, and 7.5% (*v*/*v*) acetic acid).

### 4.6. Cell Cytotoxicity Test of SWP Oil and Protein

Toxicity testing of oil and proteins from silkworm pupae was performed by using MTT (3-(4,5-dimethylthiozol-2-yl)-2,5-diphenyltetrazolium bromide) assay method. Human immortalized non-tumorigenic keratinocyte cell line HaCaT was used in this study. The cells were cultured in Dulbecco’s Modified Eagle’s medium (DMEM) and supplemented with 10% Fetal bovine serum (FBS). The cells were incubated in a humidified 5% CO_2_ incubator at 37 °C. The cells in each well of 96 well plates were treated with extracted lipid and protein from the silkworm pupae and incubated for 48 h. Then, a yellow MTT dye solution was added to the mixture. Incubation was continuously performed for 4 h at 37 °C and 5% CO_2_. The media were decanted and washed with phosphate-buffered saline solution (PBS). The produced formazan salts were dissolved with 20% SDS and its concentration was measured in a microplate reader at 570 nm. The viability of the treated cells was expressed as a percentage relative to the viability of control vehicle-treated cells. Each experiment was performed in triplicate and independently repeated at least three times.

### 4.7. In Vitro Skin Moisturizing Test

In vitro skin moisturization test of the extracted oil and protein from silkworm pupae was done by using 6-months-old back pig skin as described in Kassakul et al. [42]. The pig skin was collected from market, Bangkok, Thailand. Fat layer was removed and cut into 3 × 3 cm piece. The prepared pig skin was incubated at room temperature (27 ± 1 °C) with 50–60% RH at least 30 min before use. The SWP oil and 1 and 2% (*w*/*v*) of SWP protein in DI water were used to investigate their moisturizing efficacy and compared with commercial moisturizers: 3% glycerol, 3% propylene glycol, 3% butylene glycol, 0.2% hyaluronic acid, 3% hydrolyzed collagen and olive oil. The moisture content of the pig skin before testing was measured as base line moisture. Then, 60 µL of each sample was applied to the skin. The moisture content was measured at 10, 20 and 30 min after application by using DermaLab^®^ Series SkinLab Combo with Moisture pin probe. The experiment was done in triplicate. Skin moisturizing efficacy was calculated as:

Skin moisturizing efficacy (%) = ((At − A0)/A0) × 100(1)

where, At = skin moisture at a specified time and A0 = skin moisture at the base line.

### 4.8. Statistical Analysis

All experiments were done in triplicate and all results are presented as mean ± SD. The statistical analyses of collected datas were performed using SPSS version 19.0 (IBM). One-way analysis of variance (ANOVA) and multiple comparisons via Tukey’s test were performed to analyze the difference among data. Statistical differences were considered to be significant at *p* < 0.05.

## 5. Conclusions

The highest yields of oil and protein were obtained from 18 h extraction presenting 8.24% and 8.41% *w*/*w*, respectively. Fatty acid analysis of silkworm pupae oil as clear yellow viscous liquid was rich in linolenic acid 37.81%, oleic acid 28.97% and palmitic acid 21.27%. The TPP could extract and concentrate silkworm pupae protein showing the major bands in SDS-PAGE at around 51, 70, 175 and over 175 kDa. The protein comprised of various amino acids contributing to moisturization property such as aspartic acid, glutamic acid, glycine and serine. The silkworm oil and protein showed low cell toxicity toward human keratinocyte. The silkworm pupae oil and protein could increase moisture on pig skin in which the 2% protein was comparable in efficacy to glycerol and higher than propylene glycol, butylene glycol and hyaluronic acid. It can be presumed that the silkworm pupae which is a by-product from sericulture could be promised as high moisturizing efficacy bio-based materials. They can be potentially used as an alternative ingredient in cosmetic industry.

## Figures and Tables

**Table 1:** Yields and protein content of extracted protein and oil from silkworm pupae.

Extraction Time (h)	Oil Yield (% *w*/*w*)	Protein Yield (% *w*/*w*)	Protein Content (mg/g Extract)
3 h	7.63 ± 0.58 ^b1^	2.56 ± 0.36 ^c2^	267.57 ± 1.69 ^a3^
6 h	7.67 ± 0.35 ^b1^	3.75 ± 1.23 ^c2^	267.36 ± 1.25 ^a3^
12 h	7.82 ± 1.01 ^ab1^	7.01 ± 0.32 ^b2^	268.34 ± 1.71 ^a3^
18 h	8.24 ± 0.21 ^a1^	8.41 ± 0.26 ^a2^	268.25 ± 1.99 ^a3^

All values are mean ± SD of three determinations. Values in the same column with different superscript letters are significantly different (*p* ≤ 0.05). Different superscript numbers indicate the group of statistical analyses.

**Table 2:** Fatty acid composition of silkworm pupae oil.

Fatty Acids	% Content (*w*/*w*)
Lauric acid (C12:0)	0.13 ± 0.01
Myristic acid (C14:0)	0.49 ± 0.01
Palmitic acid (C16:0)	21.27 ± 0.05
Stearic acid (C18:0)	6.60 ± 0.09
Oleic acid (C18:1)	28.97 ± 0.13
Linoleic acid (C18:2)	4.73 ± 0.21
Linolenic acid (C18:3)	37.81 ± 0.34

All values are mean ± SD of three determinations.

**Table 3:** Physicochemical properties of silkworm pupae oil.

Properties	Values
Refractive index (30 °C)	1.464 ± 0.001
Viscosity (cP)	533.73 ± 2.33
Appearance	Clear viscous oil
Color	Yellow
L*	55.56 ± 0.16
a*	5.37 ± 0.16
b*	23.07 ± 0.08
Saponification value (mg KOH/g oil)	191.51 ± 0.22
Iodine value (g I_2_/100 g oil)	119.37 ± 0.67
Acid value (mg KOH/g oil)	0.94 ± 0.04
Peroxide value (mEq O2/kg oil)	2.00 ± 0.01
HLB value	5–6

All values are mean ± SD of three determinations.

**Table 4:** Amino acid content of silkworm pupae protein.

Amino Acids	Type Of Amino Acids	Content (g/100 g)
Glutamic acid	polar, negatively charged	2.26 ± 0.08
Aspartic acid	polar, negatively charged	2.78 ± 0.04
Histidine	polar, positively charged	0.38 ± 0.00
Arginine	polar, positively charged	1.78 ± 0.02
Lysine	polar, positively charged	1.48 ± 0.04
Cysteine	polar, uncharged	0.08 ± 0.00
Tyrosine	polar, uncharged	1.70 ± 0.11
Serine	polar, uncharged	2.05 ± 0.07
Glycine	polar, uncharged	2.39 ± 0.04
Threonine	polar, uncharged	1.33 ± 0.01
Alanine	non polar	1.35 ± 0.02
Proline	non polar	1.40 ± 0.12
Valine	non polar	1.26 ± 0.10
Methionine	non polar	0.83 ± 0.04
Isoleucine	non polar	0.97 ± 0.06
Leucine	non polar	1.68 ± 0.03
Phenylalanine	non polar	1.40 ± 0.01

## Data Availability

Not applicable.
